# Temporal Trends of HLA, CTLA-4 and PTPN22 Genotype Frequencies among Type 1 Diabetes in Continental Italy

**DOI:** 10.1371/journal.pone.0061331

**Published:** 2013-04-17

**Authors:** Marialuisa Spoletini, Simona Zampetti, Giuseppe Campagna, Lidia Marandola, Marco Capizzi, Raffaella Buzzetti, for the IMDIAB Study Group

**Affiliations:** 1 Department of Experimental Medicine, University of Rome “Sapienza”, Rome, Italy; 2 Department of Endocrinology, University of Campus Bio-Medico, Rome, Italy; University of Michigan Medical School, United States of America

## Abstract

The incidence of type 1 diabetes has, progressively, increased worldwide over the last decades and also in Continental Italian population. Previous studies performed in northern European countries, showed, alongside a general increase in the disease incidence, a decreasing frequency of the highest risk HLA genotype in type 1 diabetes populations, thus emphasizing the role of environmental factors. The aim of the study was to evaluate whether a decreasing trend of high risk HLA, CTLA-4 and PTPN22 genotypes would be present in type 1 diabetes subjects of Continental Italy, a country considered at low incidence of the disease compared to northern European populations. N = 765 type 1 diabetes patients diagnosed from 1980 to 2012 in Lazio region were included. For HLA, CTLA4 and PTPN22 temporal trend evaluation, subjects were subdivided into groups of years according to age at diagnosis. All subjects were typed for HLA-DRB1 and DQB1 by a reverse line blot. The CT60 polymorphism of the CTLA4 and C1858T of the PTPN22 gene were genotyped using ABI PRISM 7900HT (n = 419 and n = 364 respectively). HLA genotypes were divided in high, moderate and low risk categories. The proportion of the HLA risk categories was not statistically different over the three decades in subjects with age of onset <15 years and ≥15 years. The genotype distribution of CT60 polymorphism of CTLA4 gene did not show any change in the frequencies during time. The analysis of the PTPN22 C1858T variant revealed, instead, that the frequency of CT+TT susceptibility genotypes decreased during time (23.9% vs 13.6%, p = 0.017). We can hypothesize that the pressure of the diabetogenic environment could be milder and therefore not sufficient to reduce the need of a strong genetic background (HLA) “to precipitate” diabetes; the increased pressure of the environment could have, instead, some effects on minor susceptibility genes in our population.

## Introduction

The incidence of type 1 diabetes has increased progressively over the last half century [1] especially in young children. In Continental Italian population (Lazio region), characterized by a low incidence of the disease, we observed that the type 1 diabetes incidence has doubled in the 2004–2009 period compared to the 1989–1993 period, considering the 0–14 year age group [2]. The disease is a complex disorder caused by multiple genes, which interact with environmental factors. The genetic susceptibility will determine the probability of an unwanted outcome to the initial exposure but some environmental factors may influence the rate of progression [1]. The major genes responsible for at least 50% of the type 1 diabetes genetic component are human leukocyte antigen (HLA) linked [3]. As in the other Caucasian populations, the DRB1^*^03-DQB1^*^0201/DRB1^*^04-DQB1^*^0302 genotype confers the highest risk also in Italy where, however, only 1% of the general population carries this genotype compared to 2.5% of the general population of northern European countries [4]. Other polymorphisms have been reported to be associated with type 1 diabetes, among those the Cytotoxic T Lymphocyte–Associated Antigen-4 (CTLA-4) and in the Protein Tyrosine Phosphatase Non-receptor type 2 (PTPN22) genes, [5]–[7]. Some studies, performed in northern European countries or in patients of Anglo-Saxon origin characterized by a high incidence of the disease [8] showed a decreasing frequency of the high risk HLA genotype in type 1 diabetes patients over the last decades [9]–[12], thus emphasizing the role of environmental factors. The aim of the present study was to evaluate whether a decreasing trend of high risk HLA, CTLA-4 and PTPN22 genotype distributions would be present in Continental Italian population.

## Methods

Subjects for this study include n = 765 type 1 diabetes patients diagnosed from 1980 to 2012 recruited by participating centers of the IMDIAB group in Continental Italy (Lazio region). The diagnosis of type 1 diabetes was based on the American Diabetes Association (ADA) classification criteria [13].

Our research involved human participants and has been approved by the “Sapienza” University ethical committee and a written informed consent was obtained from all participating subjects. Written inform consent has been obtained from parents or legal guardian for all the children participating to the study. All clinical investigation have been conducted according to the principles expressed in the Declaration of Helsinki.

All subjects were Caucasians with parents of Italian origin. The age at diagnosis ranged from 1 to 49 years (12.7±8.9). Subjects were sub-divided into two categories: <15 years of age at diagnosis and ≥15 of age at diagnosis. For HLA temporal trend evaluation, all subjects were grouped by three decades of diagnosis: 1980–1989, 1990–1999, 2000–2012; for PTPN22 and CTLA4 they were divided into two groups of years of diagnosis: 1980–1995, 1996–2012 due to the smaller number of subjects evaluated, n = 419 and n = 364 respectively.

The HLA genotypes were classified in three risk categories based on the absolute risk values (AR) previously estimated in Continental Italian population (Lazio region) [14]: (1) High risk (AR = 1∶23) for DRB1^*^03-DQB1^*^0201/DRB1^*^04-DQB1^*^0302 genotype (DRB1^*^04 different from 0403, 06, 11); (2) Moderate risk (AR = 1∶150) for DRB1^*^04-DQB1^*^0302/DRB1^*^04-DQB1^*^0302, DRB1^*^03-DQB1^*^0201/DRB1^*^03-DQB1^*^0201, DRB1^*^04-DQB1^*^0302/X (X different from DRB1^*^02, 03, DRB1^*^04-DQB1^*^0302 (DRB1^*^04 not 0403, 06, 11) or DQB1^*^0602) and DRB1^*^03/X (X different from DRB1^*^02, 03, DRB1^*^04-DQB1^*^0302 (DRB1^*^04 not 0403, 06, 11) or DQB1^*^0602 genotypes; (3) Low risk (AR = 1∶1100) for the remaining genotypes.

Blood samples were collected and were stored at −20°C until used for genomic extraction of DNA. Genomic DNA was extracted using QIAamp DNA Blood Kit (QIAGEN Genomics Inc., Bothell, WA). All subjects were typed for HLA-DRB1 and DQB1 loci by polymerase chain reaction (PCR) followed by a reverse line blot assay using an array of immobilized sequence–specific oligonucleotide probes, as previously described [14]. Probes were kindly provided by Dr H. A. Erlich and T. Bugawan (not commercial kit; Roche Molecular System, Alameda USA). The CT60 polymorphism of the CTLA4 gene and the missense SNP C1858T of the PTPN22 gene were genotyped using the fluorogenic 5′ nuclease assay application of the ABI PRISM 7900HT Sequence Detection System (ABI, Foster City, CA) in [15], [16].

Comparisons of HLA, PTPN22 and CTLA-4 genotype frequencies between the cohorts over time were assessed by the Chi square or by Fischer test. A probability value of 0.05 or less was considered to be statistically significant. Based on the genotype frequencies of HLA (DRB1 and DQB1), CTLA4 and PTPN22 in Italian type 1 diabetes population, we should be able to identify statistical differences between the groups analysed with a power of 96%, 93% and 90% respectively and a p value <0.05.

## Results

The mean age at diagnosis of type 1 diabetes was 12.7±8.9 years (mean ± SD), and did not differ across decades of diagnosis. The distribution of HLA genotype categories in subjects with age of onset <15 and ≥15 years, according to the three groups of decades is shown in Table 1. We observed that the proportion of the three HLA risk genotype categories, was not statistically different between the three cohorts in subjects with age of onset <15 years and in subjects with age of onset ≥15 years. Table 2 shows the genotype frequencies of the CT60 polymorphism of CTLA-4 and PTPN22 C1858T variant in type 1 diabetes subjects sub-divided according to the year of diagnosis. The genotype distribution of CT60 polymorphism of CTLA4 gene did not show any change in the frequencies during time. Instead, the analysis of the PTPN22 C1858T variant revealed that the frequency of CT+TT susceptibility genotypes significantly decreased during time (23.9% vs 13.6%, p = 0.017).

**Table 1:** Proportion of high, moderate and low risk HLA genotypes of type 1 diabetes subjects with age of onset <15 and ≥15 years, subdivided according to year of diagnosis.

Age of onset <15 years	Year of diagnosis			
	1980–1989	1990–1999	2000–2012	p
	n = 133	n = 325	n = 79	
High risk HLA genotype†	31 (23.4)	69 (21.2)	21 (26.6)	ns
Moderate risk HLA genotypes‡	82 (61.6)	204 (62.8)	46 (58.2)	ns
Low risk HLA genotypes§	20 (15)	52 (16)	12 (15.2)	ns
**Age of onset ≥15 years**				
	n = 45	n = 115	n = 68	
High risk HLA genotype†	4 (8.9)	15 (13.0)	9 (13.2)	ns
Moderate risk HLA genotypes‡	30 (66.7)	68 (59.2)	46 (67.7)	ns
Low risk HLA genotypes§	11 (24.4)	32 (27.8)	13 (19.1)	ns

Data are expressed as number (%).

† High: DRB1*03-DQB1*0201/DRB1*04-DQB1*0302 genotype (DRB1*04 different from 0403, 06, 11).

‡ Moderate: DRB1*04-DQB1*0302/DRB1*04-DQB1*0302,DRB1*03-DQB1*0201/DRB1*03-DQB1*0201, DRB1*04-DQB1*0302/X, and DRB1*03/X (X different from DRB1*03, DRB1*04-DQB1*0302, DRB1*04 not 0403, 06, 11, or DQB1*0602/03) genotypes.

§ Low: other genotypes.

**Table 2:** Distribution of genotype frequencies of CT60 polymorphism of CTLA4 gene and of C1858T polymorphism of PTPN22 gene in type 1 diabetes subjects subdivided according to year of diagnosis.

CT60 SNP CTLA4	Year of diagnosis	
	1980–1995	1996–2012
	n = 219	n = 200
AA	46 (21.0)	46 (23.0)
AG+GG&	173 (79.0)	154 (77.0)
***C1858T SNP PTPN22***		
	n = 180	n = 184
CC	137 (76.1)	159 (86.4)
CT+TT*	43 (23.9)	25 (13.6)

Data are expressed as number (%).

& AG+GG vs AA p =  ns.

* CT+TT vs CC p = 0.017.

## Discussion

In the present study we did not observe any significant temporal change in HLA class II genotype distributions, including the highest risk DRB1^*^03-DQB1^*^0201/DRB1^*^04-DQB1^*^0302, over the last decades, both in type 1 diabetes children <15 years and in adult subjects, alongside a general increase in the disease incidence. In Continental Italy (Lazio region) type 1 diabetes incidence doubled in the years 2004–2009 (15.68 new cases per 100,000 per year <15 years of age with a peak in 2004∶17.3 for 100,000) compared to 1989–1993 and to 1990–1999 periods of time (7.9 and 8.8 new cases per 100,000 per year <15 years of age respectively) [2].

According to our results, a recent study showed no changes in HLA genotype frequencies in a large sample of type 1 diabetes subjects with age at onset ≤20 from Germany and Austria [17]. They observed that the highest risk HLA genotype was associated with a lower age at onset, as previously described in our population [18].

This finding is, apparently, in contrast with a decreasing trend of the highest risk HLA genotype observed, over the last decades, in northern European countries [9], [10], [11], [12], [19], [20] as well as in Australia, where a “predominantly Anglo-Celtic European population” was evaluated, and in United States [12] (Table 3).

**Table 3:** Temporal trends of high risk HLA genotype distribution in type 1 diabetes subjects from different countries.

Authors	Country	High risk HLA genotype	Age at diagnosis	n	≤1979	1980–1989		1990–1999	≥2000		p
			(years)		%	%		%	%		
									
Spoletini M et al.	Italy	DRB1*03-DQB1*0201/DRB1*04-DQB1*0302	<15	537		23.4		21.2	26.6		ns
Awa WL et al. [17]	Germany	DRB1*03-DQB1*0201/DRB1*04-DQB1*0302	≤20	1445				24.8			ns
Hermann R et al. [10]	Finland	DRB1*03-DQB1*0201/DRB1*04-DQB1*0302	<15	736	25.3			18.2			0.007
Lindehammer SA et al. [20]	Sweden	DQA1*0501-DQB1*0201/DQA1*0301-DQB1*0302	<18	943		35.6		36.8	19.1		<0.0001
Gillespie KM et al. [9]	UK	DRB1*03-DQB1*0201/DRB1*04-DQB1*0302	≤15	776	47			35			0.003
Fourlanos S et al. [12]	Australia	DRB1*03/DRB1*04	<18	462	79**	47		37	28		<0.0001
Steck AK et al. [19]	USA	DRB1*03/DRB1*04-DQB1*0302	≤18	4075	48			35*			<0.0001
Vehik K et al. [11]	USA	DRB1*03-DQB1*02/DRB1*04-DQB1*03	≤17	364	39				28		0.05

* 1995–2006.

** 1950–1969.

Studies performed in northern European populations demonstrated the same decreasing trend of the highest HLA susceptible genotype in children <18 years, although this frequency decreased from 35.6% in the decade 1980–1989 to 19.1% in 2000- in Swedish population [20], while decreased from 25.3% to 18.2%, in the same period of time, in the Finnish population [10]. Gillespie et al. [9] observed a significant decreasing frequency of the highest risk HLA genotype DR3-DQ2/DR4-DQ8 over decades in children ≤15 years of age, stressing the concept that a major environmental effect could have been accelerated the type 1 diabetes onset, thus diluting the concentration of high risk HLA susceptible genotypes in type 1 diabetes population. Fourlanos et al. showing a significant decreasing trend of the highest risk HLA genotype from 79% in 1950–1969 to 28% in 2000–2005 [12], in presence of an increase in type 1 diabetes incidence, concluded that changing environmental conditions have increased the chance to get the disease.

Vehik K et al [11], showed that the frequency of the DRB1*03-DQB1*02/DRB1*04-DQB1*03 was higher (39%) in type 1 diabetes children both from Hispanic and non Hispanic origin diagnosed during the 1978–1988 period than in those diagnosed during 2002–2004 (28%). The Authors concluded that the increasing environmental exposure is now able to trigger type 1 diabetes in subjects who are less genetically susceptible. This result was confirmed by two larger data sets of mainly Caucasian subjects (86.4%) with a minority of Hispanic and African American, still from USA, where there appeared a significant decrease of the highest risk DR3/DR4-DQB1*0302, over time, together with an increased percent of other HLA genotypes without HLA-DR3 or DR4 [19].

The reason why the highest HLA susceptible genotype tends to be constant in some populations while decreasing in others is not clear.

The two groups of populations **(**Italian and German vs the others) mainly differ for a) type 1 diabetes incidence, b) “ab initio” (early 80′) frequency of the HLA class II genotypes in type 1 diabetes populations, both evidences being related, at least in part.

The main difference between the two groups of populations regards the incidence of the disease, Italian [2] and German [21] populations being characterized by a moderate/low incidence (approximately 15 new cases per 100,000 per year <15 years) compared to the other countries characterized by a high incidence of the disease (new cases per 100,000 per year <15 years: Finland 64.2, Sweden 39.6, UK 26.4, USA 26.4 and Australia 23.1 [22], [23], [8], [24], [25].

Then, in Italian and German populations, the DRB1^*^03-DQB1^*^0201/DRB1^*^04-DQB1^*^0302 frequency, in type 1 diabetes population, was around 25% in the 80′ compared to >35% of that reported in the northern European countries at that time, except Finland. This finding reflected the low frequency of DRB1^*^03-DQB1^*^0201/DRB1^*^04-DQB1^*^0302 in the general populations of Italy and German and could be, at least in part, the reason why the incidence of the disease has been low so far in these countries, assuming that the Odds Ratio (ORs) for every specific genotype tended, at that time, to be constant in all Caucasian populations.

Over the past few decades we assisted at a decreasing frequency of the highest HLA susceptible genotype in the diabetes population of countries with a high incidence of the disease, but not in those with a low incidence. Based on these evidences we can speculate that different environmental factors in various populations could differently influence the penetrance of HLA genes. Thus, populations with a lower incidence could be at an earlier stage of the natural evolution of diabetes history; the pressure of the diabetogenic environment could be milder and still “polarized” to the highest HLA genotype, as not sufficient to reduce the need of a strong genetic background (HLA) “to precipitate” diabetes. A logical consequence is that the HLA class II genotypes ORs appear now to be different in the different populations.

Viral infections [26], physical inactivity, excess of food intake or excessive hygiene may contribute to the increase incidence of type 1 diabetes [27]. It is conceivable that a decreased microbial load in early life may have a major impact on the programming of the immune system, particularly the gut-associated lymphoid tissue [28].

Environmental factors could either modify the penetrance of susceptibility genes, or, as triggering factors, could contribute directly to the incidence. It has been hypothesized that changes in penetrance might be linked to patterns of childhood immunization, but this has yet to be confirmed [29]. Environmental exposures to dietary antigens and microbes could be implicated in the increasing incidence of type 1 diabetes. However, no single pathogenic environmental agent has been identified that explains all cases [30].

Due to the limited sample size of subjects, we sub-divided the genotype distribution of CTLA4 and PTPN22 into two groups: 1980–1995 and 1996–2012 according to the year of diagnosis of type 1 diabetes. As well as for HLA genotypes, also the distribution of CT60 polymorphism of CTLA4 gene did not show any change in the frequencies during time, as previously demonstrated [10].

Conversely, we observed that the frequency of CT+TT susceptibility genotypes of PTPN22 gene decreased during time. For our knowledge this is the first study to analyze the PTPN22 genotype frequency in type 1 diabetes during decades. The risk conferred by this genotype to the disease is quite small (OR = 2.48) [31] compared to that conferred by HLA DRB1*03-DQB1*0201/DRB1*4-DQB1*0302 in our population OR = 24.7 [14]. The increased pressure of the environment could have some effects on minor susceptibility genes and still not on the major HLA susceptibility component in our population.

Our study has some limitations, the most important one is due to the small number of subjects who took part to this study in the last decade compared to the previous ones. Nevertheless our data contribute to clarify that the increase in type 1 diabetes over the last decades might be explained by a complex interactions between genes and environmental risk factors which may differ in the different populations. The epidemiology of type 1 diabetes suggests that varying gene–environment interactions are likely triggering and/or accelerating the autoimmune destruction of β-cells leading to complete insulin deficiency [32]. When the T susceptibility allele of PTPN22 gene is not present the environmental factors could have a predominant role in the type 1 diabetes pathogenesis.

We can hypothesize that also epigenetic regulation could be one way to explain the rapid increase in incidence and could be a central mechanism by which environmental factors can influence the development of type 1 diabetes [33].
